# Program of the Second Union Meeting of the Washington City Dental Society and the Maryland State Dental Association, at the Royal Arcanum Hall, Baltimore, Md.

**Published:** 1895-05

**Authors:** 


### PROGRAM OF THE SECOND UNION MEETING OF THE WASHINGTON CITY DENTAL SOCIETY AND THE MARYLAND STATE DENTAL ASSOCIATION, AT THE ROYAL ARCANUM HALL, BALTIMORE, MD.

---

MORNING SESSION, WEDNESDAY APRIL 17TH, AT 10 A. M.

Cyrus M. Gingrich, D. D. S., of Baltimore, Chairman of Joint Committee, will open the meeting.

C. C. Harris, M. D., D. D. S., of Baltimore, President of the Maryland State Dental Association, will make the Address of Welcome.

J. Roland Walton, D. D. S., of Washington, President of the Washington City Dental Society, will respond.

Roport of Committee on Publication, Voluntary Essays and Dental Legislation. Williams Donnally, D. D. S., Chairman, of Washington.

Essay, "Pyorrhœa Alveolaris," B. F. Arrington, M. D., D. D. S., Goldsboro, N. C.

AFTERNOON SESSION, AT 2 P. M.

Report of Committee on Dental Education, Literature and Dental Nomenclature. M. F. Finley, D. D. S., Chairman, of Washington.

Essay, "Dental Education," A. W. Sweeny, D. D. S., of Washington,

Report of Committee on Orthodontia and Dental Appliances. Jos. Roach, D. D. S., Chairman, of Baltimore.

EVENING SESSION AT 8 P. M.

Essay, "Bacteria of the Mouth," Dr. Wm. H. Welsh, Professor of Pathology, Johns Hopkins University, Baltimore.

Essay, "Nitrous Oxide as an Anæsthetic," Dr. Geo. T. Kemp, of Johns Hopkins University.

Report of Committee on Pathology and Therapeutics. E. P. Keech, M. D., D. D. S., Chairman, of Baltimore.

Report of Committee on Operative Dentistry and Surgery. W. St. Geo. Elliott, M. D., D. D. S., Chairman, of New York.

MORNING SESSION, THURSDAY, APRIL 18th, AT 10 A. M.

Report of Committee on Anatomy, Physiology and Histology. D. Elmer Wiber, D. D. S., Chairman, of Washington.

Essay, "Scientific Root Filling and Ideal Crown and Bridge Work," Nelson T. Shields, D. D. S., of New York.

Report of Committee on "Crown and Bridge Work." H. M. Schooley, D. D. S., Chairman, of Washington.

AFTERNOON SESSION AT 2 P. M.

Essay, "The Benefits of Laws Regulating the Practice of Dentistry," L. Ashley Faught, D. D. S., of Philadelphia.

Report of Committee on "Mechanical Dentistry." A. J. Volck, Ph. D., M. D., D. D. S., Chairman, of Baltimore.

Banquet at 8 P. M.

### CLINICS.

Dr. W. B. Finney, Baltimore, Md., will insert contour gold filling.

Dr. H. B. Noble, Washington, D. C., will exhibit Models of Irregularities and their Appliances.

Dr. Nelson T. Shields, of New York, will make a piece of Bridge-Work; will also show practical cases in the mouths of patients from New York; will exhibit a Porcelain Bridge ready for insertion, and will afterwards place it in the mouth of his patient."

Dr. F. F. Drew, of Baltimore, Md., will exhibit a case of correcting an irregularity by the Masking Method.

Dr. Wm. Shaw Twilley, of Baltimore, Md., will show a method of placing and replacing porcelain faces in Crown and Bridge Work.

Dr. N. T. Shields, New York, will demonstrate his method of filling difficult root canals to their apices.

Dr. L. B. Wilson, Cumberland, Md., will demonstrate his method of making crowns and bridge work by the electro-deposition.

Dr. David Genese, Baltimore, Md., will present a case showing treatment and its results in bridge work, in gold and the same in vulcanite, with treatment and extraction of nerve under a new method.

Dr. Chas. B. Munson, Washington, D. C., will make and demonstrate the Jackson wire crib for regulating teeth.

Dr, C. C. Carroll, Meadville, Pa., will give a clinical lecture on Aluminum in Dentistry.

Dr. J. L. Wolf, Washington, D. C., will fill a tooth with amalgam, securing additional anchorage by means of oxy-phosphate.

Dr. A. Lee Penuel, Leesburg, Va., will insert a contour gold filling.

### EXHIBITS.

The S. S. White Dental Mfg. Co., Phila., Pa., will exhibit Electrical Appliances and Novelties of recent invention.

The Hollingsworth System of Crowns and Bridges will be demonstrated.

The Harvard Co., of Canton, Ohio, will display a Dental Chair and Cabinet.

The Horlick Food Co., of Racine, Wis., will exhibit their Malted Milk, and furnish members with samples of same.

J. W. Ivory, of Philadelphia, Pa., will display a full line of his Dental Specialties.

The Chas. H. Phillips Chemical Co., New York, will make an exhibit of their Antacid Preparation—Phillips Milk of Magnesia and explain its uses.

The Medical Novelty Co., New York, will make an exhibit of Borine and will distribute sample bottles of same to the profession.

The Diamond Tooth and Tool Co., New York, will make an exhibit of Burs.

TOASTS AT THE SECOND UNION DINNER.

F. J. S. Gorgas, M. D., D. D. S., Baltimore, Presiding.

E. P. Keech, M. D., D. D. S., Baltimore, "Dental Profession."

L. C. F. Hugo, D. D. S., Washington. D. C., "The Dental Limbo."

L. Ashley Faught, D. D. S., Philadelphia, Pa., "The National Association of Dental Examiners."

B. Holly Smith, M. D., D. D. S., Baltimore, "Union Meetings."

A. W. Sweeny, D.D.S., Washington, D. C., "Modern Dentistry."

H. B. Noble, D. D. S., Washington, D. C., "Dental Societies and Society Work as a Means of Education."

N. T. Shields, D. D. S., New York, "The Future of Dentistry."

Richard Grady, M. D., D. D. S., Baltimore; A. G. Cockerille, Washington, D. C.; A. J. Volck, M.D., D.D.S., "Dental Literature."

